# Laparoscopic versus open splenectomy in non-traumatic pediatric patients: a systematic review and meta-analysis

**DOI:** 10.1007/s00383-025-06208-2

**Published:** 2025-10-17

**Authors:** Alma Sato, Marios Alogakos, James W. F. Burns, Megan Roberts, Dafni-Stravroula Grammenou, Ebaney Ghotra, Emily E. Lugard, Charbel Chidiac, Christian Aloysius Than, Hayato Nakanishi, Shaun M. Kunisaki

**Affiliations:** 1 University of Nicosia Medical School, University of Nicosia, 2417 Nicosia, Cyprus; 2 St George’s University of London, London, SW17 0RE UK; 3 Manchester University NHS Foundation Trust, Manchester, M13 9WL UK; 4 European University Cyprus Medical School, 6 Diogenous Str., Egkomi, 2404 Nicosia, Cyprus; 5 Department of Surgery, Division of General Pediatric Surgery, Johns Hopkins University School of Medicine, Baltimore, MD 21287 USA; 6 School of Biomedical Sciences, The University of Queensland, St Lucia, Brisbane, 4072 Australia; 7 Cambridge University Hospitals NHS Foundation Trust, Addenbrookes Hospital, Cambridge, CB2 0QQ UK; 8 Department of Surgery, Mayo Clinic, Rochester, MN 55905 USA

**Keywords:** Spleen, Laparoscopic, Splenectomy, Open splenectomy, Pediatric, Hematologic disorders

## Abstract

**Background:**

Laparoscopic splenectomy (LS) is the preferred surgical approach for pediatric patients requiring splenectomy, though gaps remain regarding spleen size impact on outcomes and conversion rates, especially in children with hematologic disorders.

**Methods:**

A comprehensive literature search was conducted across PubMed, Medline, CINAHL, Embase, and Cochrane Library, from inception to January 2025, following PRISMA guidelines and registered with PROSPERO (CRD42025644989). Statistical analysis was performed using a random-effects model.

**Results:**

The initial search yielded 216 studies, 19 studies with 1898 pediatric patients included. Of those, 1202 underwent LS and 696 open splenectomy (OS). LS was associated with shorter hospital stay (MD: −1.59 days, 95% CI: −2.18 to −1.00, *P* < 0.00001), faster initiation of oral feeding (MD: −0.68 days, 95% CI: −1.07 to −0.29, *P* = 0.0006), fewer transfusions (OR = 0.38, 95% CI: 0.23 to 0.62, *P* = 0.0001), and conversion to OS in 2.7% of cases. OS showed shorter operative time (MD: 60.4 min, 95% CI: 37.4 to 83.4, *P* < 0.00001), and improved accessory spleen removal (OR: 1.91, 95% Cl: 1.02 to 3.57, *P* = 0.04).

**Conclusion:**

Our findings support LS as a safe and effective technique in pediatric hematologic patients and provide updated evidence to guide surgical decision-making.

**Supplementary Information:**

The online version contains supplementary material available at 10.1007/s00383-025-06208-2.

## Introduction

In the last decade, over a thousand splenectomy cases were performed in the United States for pediatric patients with hematologic pathology [1]. When medical treatment fails, splenectomy becomes a cornerstone in management for hematologic disease, focal splenic lesions, and trauma [2, 3]. Existing literature reports the splenectomy benefits in alleviating anemia and thrombocytopenia, preventing splenic rupture, and improving quality of life [4–6]. However, recent technical innovations and increasing aptitude in minimally invasive surgery (MIS) have prompted discussion on the optimal approach to pediatric splenectomy [7].

Open splenectomy (OS) has traditionally been the standard surgical approach [8]. Wu et al. [9] argue that OS provides better exposure and control in cases of large spleens, portal hypertension or significant bleeding risks, while minimizing the likelihood of injuring the surrounding organs. Furthermore, larger specimen extraction is streamlined from concurrent larger incisions [10]. Nonetheless, OS has drawbacks, including longer hospital stay and abdominal scarring. In an effort to optimize patient outcomes, exploration of alternative approaches to performing splenectomy has arisen [11].

Laparoscopic splenectomy (LS) was first introduced for the adult population by Delaitre et al. in 1991 and later introduced to the pediatric population by Tulman et al. in 1993 [12, 13]. As illustrated in the National Surgical Quality Improvement Program Pediatric (NSQIP-P) data, favorable perioperative profiles have driven surgeons to increasingly prefer LS for non-traumatic splenic disease [4, 5, 14]. Notably, several investigators have suggested that LS could improves recovery, decreases complications, and achieves superior cosmetic outcomes [6, 15, 16]. Despite these benefits exist, the adoption of LS in pediatric splenectomy remains a topic of ongoing debate, especially in cases involving large spleens or complex hematologic pathology [4, 6].

Despite the Society of American Gastrointestinal and Endoscopic Surgeons (SAGES) guidelines recommending LS as the preferred approach for centers with advanced surgical expertise [17], no universal consensus defines it as the standard of care, particularly for patients with large spleens associated with hematologic disease. Several existing reviews have demonstrated the safety and efficacy of LS in pediatric patients; however, many of these studies include heterogeneous cohorts with both hematologic and traumatic indications, limiting conclusions for hematologic disease [15, 18]. Furthermore, earlier meta-analyses have primarily focused on a limited set of perioperative outcomes such as operative time, blood loss, length of stay, and certain complications, as well as other clinically significant measures, including spleen size and weight, transfusion requirements, conversion rates, a comprehensive complication profile, mortality, and time to initiation of postoperative feeding [15, 18]. Given the growing adoption of LS and the persistent lack of guidelines specifically addressing splenectomy in pediatric hematologic conditions involving large spleens, further focused investigation is warranted.

The objective of this study was to evaluate perioperative outcomes of LS in pediatric patients with hematologic disorders, including spleen size and weight, conversion rates, transfusion requirements, complications, mortality, and time to feeding. With advancements in MIS but limited guideline refinement, further investigation is warranted. This meta-analysis re-evaluates the safety and efficacy of LS compared with OS to optimize management of non-traumatic splenic disease in pediatric patients and provide an updated evidence base for surgical decision-making.

## Methods

### Data sources and search strategies

A comprehensive literature search was conducted across multiple databases, including Ovid MEDLINE, Embase, Cochrane Library, and CINAHL from their inception to January 29, 2025. The search strategy was designed and conducted by an experienced librarian with input from the study’s principal investigator, using a combination of controlled vocabulary and keywords focused on laparoscopic versus open splenectomy in pediatric patients for non-traumatic splenic diseases. The search strategy is detailed in Supplemental Item 1. This study was conducted in compliance with the Preferred Reporting Items for Systematic Reviews and Meta-analyses (PRISMA) guidelines [19] and was prospectively registered with PROSPERO (CRD42025644989).

### Eligibility criteria and quality assessment

Eligible studies were cohort studies that met all the following inclusion criteria: (1) pediatric patients aged ≤18 years; (2) focused on non-traumatic splenic disease as indications for splenectomy, including hematological and focal lesions; (3) compared laparoscopic splenectomy and open splenectomy; and (4) reported primary outcomes. Case reports, case series, abstracts, conference abstracts, reviews, and articles that were not reported in English were excluded from the study. Additionally, this study excluded cases of emergency splenectomy.

The classification of LS and OS groups was determined based on intention-to-treat analysis. Article screening were conducted by two of six independent assessors for each paper (MR, EL, EG, JB, MA, or DG). Each article required agreement from at least two different assessors to be included. Data extraction was conducted by all six independent assessors (MR, EL, EG, JB, MA, or DG). The quality of each study was independently evaluated by two authors (JB, MR) using the Risk of Bias in Non-Randomized Studies of Interventions (ROBINS-I) [20]. Any disagreements in screening and quality assessments were adjudicated by a third author (AS) and discussed with co-authors as necessary. The methodology and results of the quality assessment of all included studies are summarized in Supplemental Item 2.

### Extracted outcomes

Intraoperative outcomes included conversion to open surgery, operative time, intraoperative estimated blood loss (EBL), concomitant cholecystectomy, accessory spleen presence, and accessory spleen removal. Postoperative outcome included postoperative length of hospital stay, overall complication rates, transfusion rates, initiation to feed, mortality, and ICU admission. Transfusion rates are defined as the number of blood transfusion units required per patient during and after splenectomy.

### Statistical analysis

The pooled means and proportions for our data were analyzed using a random-effects, generic inverse variance method for continuous data and the Mantel–Haenszel method for dichotomous data, which assigns the weight of each study based on its variance. This meta-analysis was restricted to studies that reported outcomes for both LS and OS within the same study, enabling a direct comparison between the two techniques (two-arm analysis). The heterogeneity of effect size estimates across the studies was quantified using the Q statistic and *I*^2^. A value of *I*^2^ of 0–25% indicates insignificant statistical heterogeneity, 26–50% low heterogeneity, and 51–100% high heterogeneity [21]. When mean and standard deviation (SD) were unavailable, the median was converted to mean using the formulas from the Cochrane Handbook for Systematic Reviews of Interventions [22]**.** If the SD was not available or extractable, the reported mean was omitted from the calculation. Data analysis was performed using RevMan software version 5.4 (Review Manager, The Cochrane Collaboration, 2020, Copenhagen, Denmark).

## Results

### Study selection and patient characteristics

The initial literature search of the electronic databases yielded 216 studies. After removing duplications and applying the inclusion and exclusion criteria, 42 studies were retained for full-text review. Nineteen unique studies involving 1898 patients were included in this meta-analysis (Supplemental Item 3). These studies were all retrospective, of which four were multicenter studies [23–26] and 15 were single-center studies [4, 7, 17, 23, 27–36]. The mean age of participants across studies ranged from 4.3 to 13.2 years, and 1175 (61.9%) patients were female. Among the studies reporting splenic pathology, the most common indications for splenectomy were hereditary spherocytosis (HS) (*n* = 385, 20.2%), thalassemia (*n* = 364, 19.2%), sickle cell disease (SCD) (*n* = 334, 17.6%), and idiopathic thrombocytopenic purpura (ITP) (*n* = 190, 10%). The baseline characteristics of the included studies are described in Table 1.

**Table 1:** Baseline and clinical characteristics of included studies

Study	Publication year	Country	Study type	Total number of participants, *N*	Number of patients in each group, *N*	Gender (Female), *N* (%)	Age, Mean ± SD (Years)	Splenectomy indications	Spleen size mean (cm)±SD	Spleen weight mean (g)±SD
Alwabari et al. [27]	2009	Saudi Arabia	Retrospective study	150	LS:30 OS: 120	60 (40.0%)	LS:7.0±2.5 OS:7.6±2.8	LS:30 SCD OS:120 SCD	LS:NR OS:NR	LS:NR OS:NR
Deng et al. [14]	2012	China	Retrospective study	57	LS:30 OS:27	19 (33%)	LS:8.0±3.0 OS:8±1.25	LS:4 HS, 17 TH, 9 ITP OS:3 HS, 20 TH, 4 ITP	LS:NR OS:NR	LS:NR OS:NR
Esposito et al. [17]	1997	Italy	Retrospective study	16	LS: 8 OS:8	NR	LS:NR OS:NR	LS:NR OS:NR	LS:NR OS:NR	LS:NR OS:NR
Esposito et al. [23]	2022	Italy	Retrospective study	121	LS: 90 OS:32	48 (39.6%)	LS:10.2±2.0 OS:10.3±2.0	LS:28 HS, 34 TH, 4 SCD 20 ITP, 4M OS:17 HS, 6 TH, 7 ITP, 1 M	LS:NR OS:NR	LS:NR OS:NR
Fachin et al. [29]	2019	Brazil	Retrospective study	35	LS: 13 OS:2	19 (54%)	LS: 4.3 OS:4.7	LS:3 HS, 10 SCD OS:5 HS, 14 SCD, 1 ITP, 1 AIHA, 1 H	LS:11.5 OS:12.4	LS:NR OS:NR
Farah et al. [28]	1997	US	Retrospective study	36	LS: 16 OS:20	21 (58.0%)	LS:10.3±3.7 OS:9.7±4.3	LS: 5 HS, 6 SCD, 4 ITP, 1 AIHA OS:3 HS, 2 TH, 7 SCD, 7 ITP, 1 AIHA	LS:NR OS:NR	LS:209.9 OS:272.9
Hassan et al. [24]	2014	Egypt, UAE	Retrospective study	32	LS: 12 OS:20	19 (59.0%)	LS:8.5±1.9 OS:8.0±1.7	LS:NR OS:NR	LS:NR OS:NR	LS:218±118.5 OS:360±299
Janu et al. [30]	1996	US	Retrospective study	61	LS: 14 OS:47	NR	LS:NR OS:NR	LS:NR OS:NR	LS:NR OS:NR	LS:800±157 OS:850±185
Khirallah et al. [31]	2017	Egypt	Retrospective study	70	LS: 35 OS:35	50 (71.0%)	LS:8.1 OS:8.42	LS: 5 HS, 18 TH, 12 ITP OS: 5 HS, 18 TH, 12 ITP	LS:14.02 OS:13.9	LS:NR OS:NR
Makansi et al. [32]	2021	Germany	Retrospective study	26	LS: 10 OS:16	17 (65.0%)	LS:13.1±3.15 OS:10.7±3.3	LS: 9 HS, 1 SC OS: 14 HS, 2 Di George	LS:15.8±4.2 OS:14±4.8	LS:NR OS:NR
Minkes et al. [4]	2000	US	Retrospective study	52	LS:35 OS:17	27 (52.0%)	LS:9.4±4.0 OS:11.8±3.75	LS: 5 HS, 20 ITP, 6 SCD/T OS: 5 HS, 4 ITP	LS:6.3±2.2 OS:48.5±3.6	LS:NR OS:NR
Moores et al. [7]	1995	US	Retrospective study	32	LS:12 OS:20	NR	LS:8.6±4.4 OS:7.3±3.18	LS: 5 HS, 1 TH, 1 SCD, 5 ITP OS: 3 HS, 1 TH, 5 SCD, 11 ITP	LS:NR OS:NR	LS:NR OS:NR
Qureshi et al. [33]	2005	US	Retrospective study	140	LS:81 OS:59	65 (46.0%)	LS:11.6±0.5 OS:10.9±0.6	LS: 50 HS OS: 22 HS	LS:NR OS:NR	LS:308±45 OS:509±110
Reddy et al. [8]	2001	US	Retrospective study	45	LS:16 OS:29	29 (64.0%)	LS:11.2±2.8 OS:8.6±2.1	LS: 6 HS, 1 SCD, 5 ITP, 3 AIHA OS: 9 HS, 6 SCD, 6 ITP, 3 AIHA	LS:NR OS:NR	LS:NR OS:NR
Utria et al. [25]	2019	US	Retrospective study	673	LS:613 OS:60	637 (94.0%)	LS:8.5±2.6 OS:8.6±2.1	LS: 118 HS, 117 SCD, 52 ITP, 51 S, 3 M, 72 O	LS:NR OS:NR	LS:NR OS:NR
Waldhausen et al. [34]	1997	US	Retrospective study	20	LS:10 OS: 10	NR	LS:10.3±3.3 OS:8.3±3.3	LS: 7 HS, 3 ITP OS: 4 HS, 2 TH, 3 ITP, 1 M	LS:NR OS:NR	LS:NR OS:NR
Wang et al. [35]	2023	China	Retrospective study	274	LS:147 OS:127	117 (42.0%)	LS:8±1.25 OS:8±1.25	LS: 13 HS, 130 TH, 4 O OS: 10 HS, 115 TH, 2 O	LS:20.2±1 OS: 20.5±0.875	LS:NR OS:NR
Yoshida et al. [36]	1995	Japan	Retrospective study	19	LS:8 OS:11	11 (68.0%)	LS:32.4 OS:28.5	LS: 6 HS, 2 ITP OS: NR	LS:NR OS:NR	LS:182±62.2 OS:145±49.7
Zhu et al. [26]	2011	China	Retrospective study	39	LS:22 OS:17	23 (59.0%)	LS:43.2 OS:44.7	LS: 10 HS, 6 ES, 6 H OS: 7 HS, 1 SCD, 1 ITP, 4 H	LS:15.8±7.9 OS:16.3±7.7	LS:NR OS:NR

*LS* Laparoscopic surgery, *N* Number of patients, *NR* Not reported, *OS* Open surgery, *SD* Standard deviation, *HS* Hereditary spherocytosis, *ITP* Immune thrombocytopenic purpura, *SCD* Sickle cell disease, *AIHA* Autoimmune hemolytic anemia, *TH* Thalassemia, *M* Malignancy, *S* Splenomegaly, *ES* Evan’s syndrome, *O* Other

### Risk of bias

Of the cohort studies judged via ROBINS-l tool, three were classified as low risk [23, 32, 35], nine as moderate risk [4, 7, 17, 24, 25, 28, 30, 33, 36], and seven as serious risk [8, 14, 26, 27, 29, 31, 34]. Serious risk was primarily attributed to confounding [8, 14, 26, 26, 27, 31, 34] due to non-randomized allocation and inadequate adjustment for key confounders such as baseline disease severity and prior treatments. Nonetheless, all studies were deemed eligible for analysis as patients appeared to represent the whole experience of the investigator. The exposure and outcomes were adequately ascertained, and the lengths of follow-up were adequate for the purposes of this study. Results of the quality assessment of all included studies are shown in Supplemental Item 2.

### Clinical characteristics

**Fig. 1:**
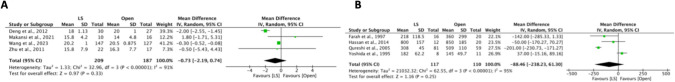
HYPERLINK "sps:id::fig1||locator::gr1||MediaObject::0" The pooled estimate of baseline clinical characteristics between laparoscopic surgery and open surgery: (**A**) Spleen size and (**B**) Spleen weight

Among the nineteen included studies, a total of 1898 patients underwent either LS (*n* = 1202, 63%) or OS (*n* = 696, 37%). Our study demonstrated no difference in the spleen size (MD = −0.7 cm, 95% CI: −2.2 to 0.7, *I*^2^ = 91%, *P* = 0.33) and weight (MD = −88.5 g, 95% CI: −238.2 to 61.3, *I*^2^ = 95%, *P* = 0.25) between the groups. Subgroup analysis on weight and size of spleen was not feasible due to limited about of data. The clinical characteristics of the included studies are summarized in Fig. 1 and Table 1.

### Intraoperative outcomes

**Fig. 2:**
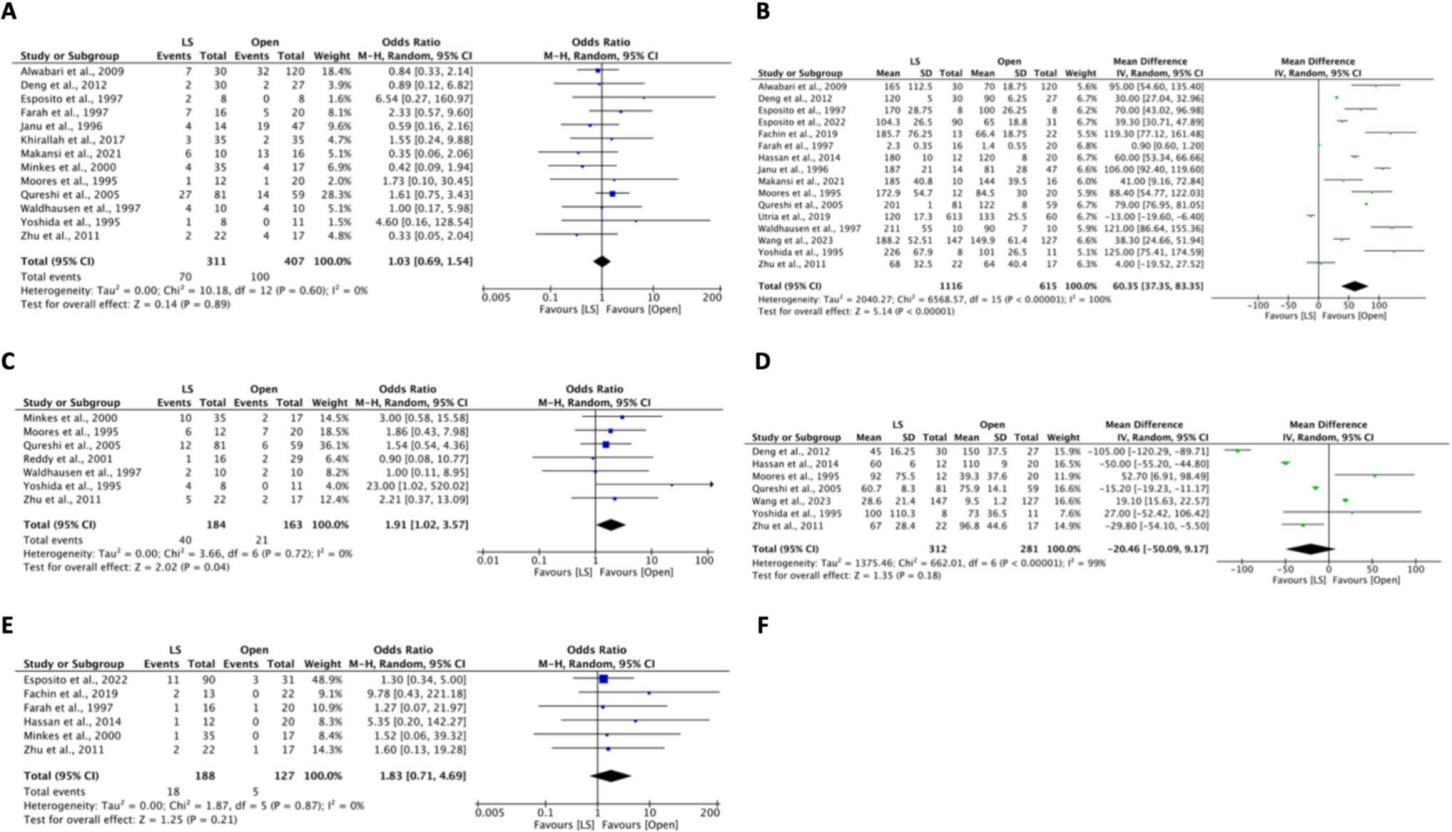
Pooled estimate of intraoperative outcomes of laparoscopic surgery and open surgery: (**A**) Concurrent cholecystectomy; (**B**) Operative time; (**C**) Accessory spleen removal; (**D**) Estimated blood loss; and (**E**) Intraoperative complications

The summary of intraoperative outcomes is described in Fig. 2. The pooled mean operative time was longer for the LS group compared to the OS group (MD = 60.4 min, 95% CI: 37.4 to 83.4, *I*^2^ = 100%, *P* < 0.00001). There was no difference in EBL (MD = −20.5 mL, 95% CI: −50.1 to 9.2, *I*^2^ = 99%, *P* = 0.18) [7, 14, 24, 26, 33, 35, 36]. The rate of accessory spleen removal was evaluated among seven studies [4, 7, 8, 26, 33, 34, 36], with a higher rate in the OS group (OR: 1.91, 95% CI: 1.02 to 3.57, *I*^2^ = 0%, *P* = 0.04). Concomitant cholecystectomy was performed in 8.3% (*n* = 100) of LS cases and 10% (*n* = 70) of OS cases, with no differences in rates (OR = 1.03, 95% CI: 0.69 to 1.54, *I*^2^ = 0%, *P* = 0.89) [4, 7, 8, 14, 17, 26–28, 30–36]. Similarly, intraoperative complications showed no differences (OR = 1.83, 95% CI: 0.71 to 4.69, *I*^2^ = 0%, *P* = 0.12) [4, 23, 24, 26, 28, 29]. Intraoperative complications included spleen rupture (*n* = 3), colon rupture (*n* = 1), and diaphragmatic tear (*n* = 1) [23, 26, 28]. Lastly, across fifteen studies, the rate of conversion to OS was 2.7% (*n* = 33) [4, 7, 8, 14, 23–28, 30–34]. Sensitivity analysis excluding studies prior to 1997 demonstrated that most outcomes remained stable. However, Accessory spleen removal no longer showed a difference between LS and OS (OR: 1.79, 95% CI: 0.85 to 3.81, *I*^2^ = 0%, *P* = 0.13) [4, 8, 26, 33], while estimated blood loss was lower in LS (MD: −35.73, 95% CI: −68.72 to −2.75, *I*^2^ = 99%, *P* = 0.03) [14, 24, 26, 33, 35]. These findings likely reflect evolving surgical techniques and perioperative management over time. Importantly, the overall trends favoring laparoscopic splenectomy remained consistent, supporting the robustness of our conclusions.

### Postoperative outcomes

**Fig. 3:**
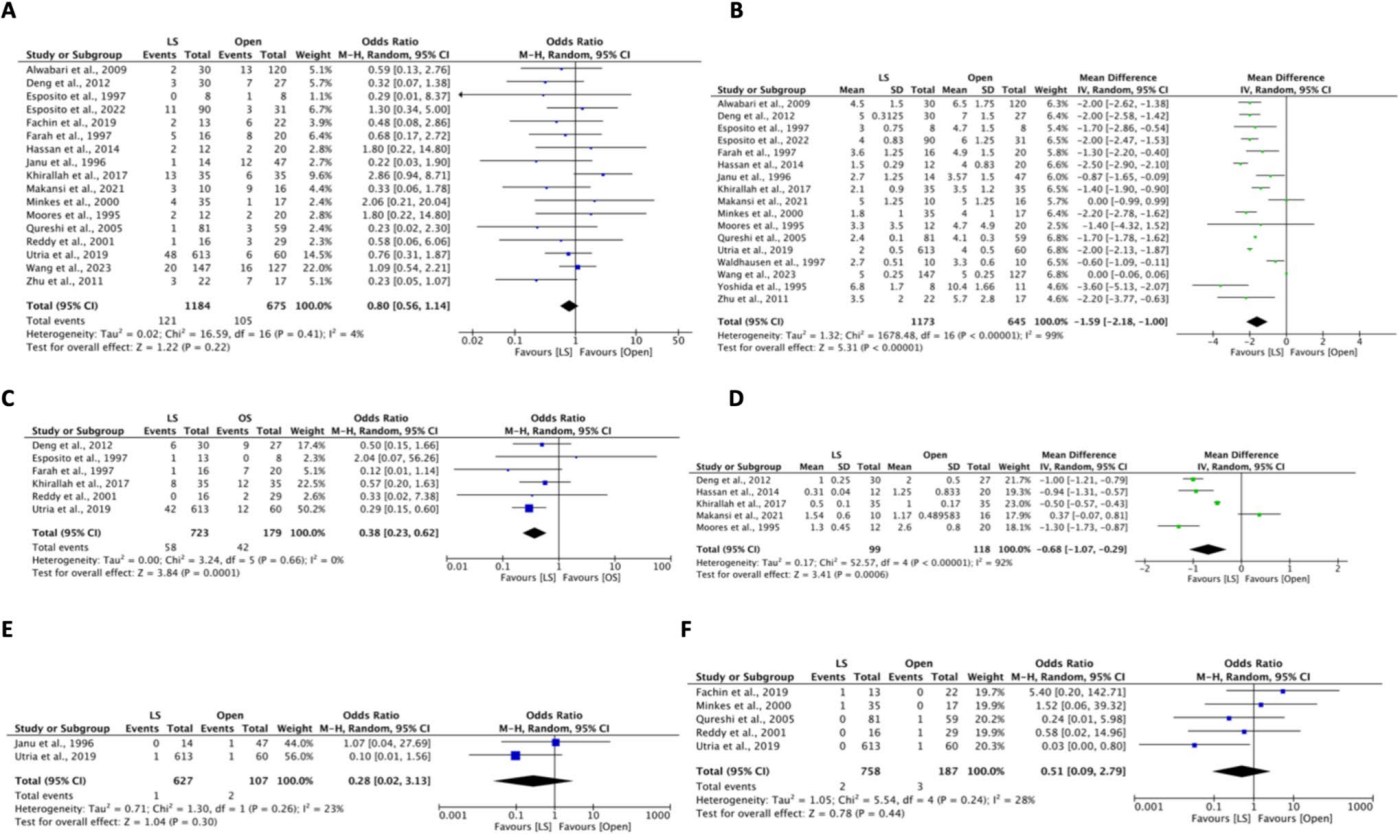
Pooled estimate of postoperative outcomes of laparoscopic surgery and open surgery: (**A**) Overall complications; (**B**) Length of hospital stay; (**C**) Transfusion; (**D**) Initiation to feed; (**E**) ICU admission; and (**F**) Mortality

**Fig. 4:**
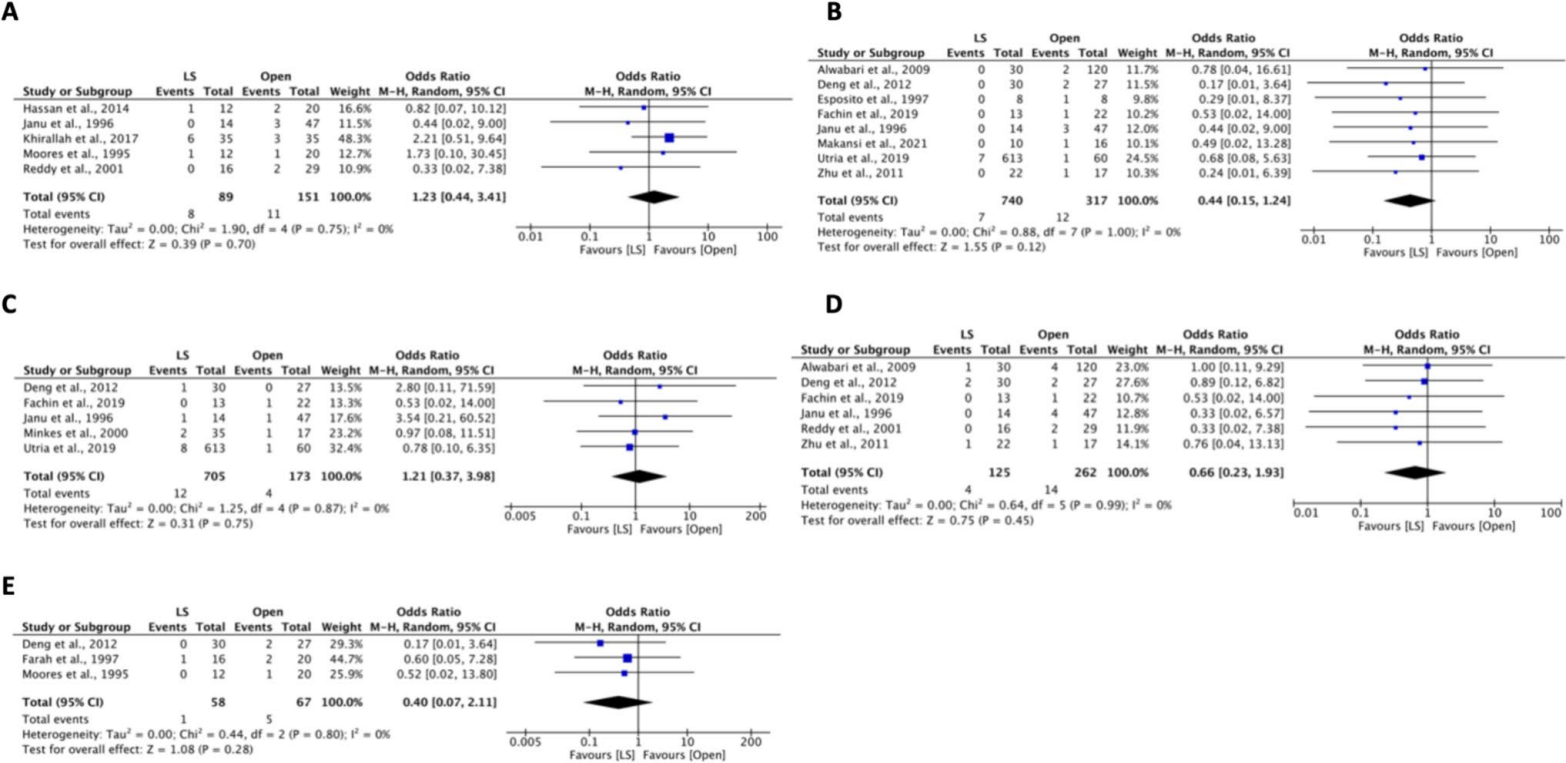
Pooled estimate of specific postoperative complications of laparoscopic surgery and open surgery: (**A**) Atelectasis; (**B**) Infection; (**C**) Pneumonia; (**D**) Postoperative fever; and (**E**) Postoperative ileus

Postoperative outcomes are shown in Figs. 3 and 4. Hospital stay was evaluated in all included studies. The LS group had a shorter hospital stay compared to the OS group (MD: −1.6 days, 95% CI: −2.2 to −1.0, *I*^2^ = 99%, *P* < 0.00001) [4, 7, 8, 14, 17, 23–36]. Additionally, the initiation to feed was faster in the LS group (MD: −0.7 days, 95% CI: −1.1 to −0.3, *I*^2^ = 92%, *P* = 0.0006) [7, 14, 24, 31, 32]. Transfusion requirement was lower in the LS group (OR = 0.38, 95% CI: 0.23 to 0.62, *I*^2^ = 0%, *P* = 0.0001) [4, 7, 8, 14, 17, 25, 28, 31, 36]. No difference was seen in overall mortality (OR = 0.51, 95% CI: 0.09 to 2.79, *I*^2^ = 28%, *P* = 0.44) [4, 8, 14, 17, 25, 26, 28–30, 33, 35] and ICU admission rate (OR = 0.28, 95% CI: 0.02 to 3.13, *I*^2^ = 23%, *P* = 0.30) [25, 30, 32]. Similarly, no differences were observed in overall complications [4, 7, 8, 14, 17, 23–36] as well as the rate of atelectasis [7, 8, 24, 30, 31], pneumonia [4, 14, 25, 29, 30], postoperative fever [8, 14, 26, 27, 29, 30], postoperative ileus [7, 8, 14, 28], and infection [14, 17, 25–32].

## Discussion

Splenectomy remains a crucial intervention for managing non-traumatic splenic diseases in pediatric patients and the choice between LS and OS has been a topic of clinical debate. This meta-analysis compared the safety and efficacy of LS and OS for this setting. Our results demonstrated that baseline clinical characteristics of patients were similar across both groups. Notably, LS was associated with a shorter hospital stay, quicker initiation of oral intake, and reduced blood transfusion requirements compared to OS. Additionally, no differences in overall complications or EBL between the two approaches.

A central concern in the selection of LS has historically been the presence of splenomegaly, which is viewed as posing technical challenges related to reduced intra-abdominal working space, complex splenic mobilization, and difficulties with specimen retrieval and hemostasis [37]. However, our findings showed no difference in spleen size or weight between LS and OS groups, and only 8 of 19 studies reported either one of these parameters, limiting the ability to draw statistically meaningful conclusions. Although massive splenomegaly is frequently mentioned in the literature, no consensus has been reached on a universal definition. Interestingly, the current definition falls in the range of 1000 to over 2000 g across studies, highlighting the need for standardized criteria and consideration of weight adjustments in small children [24, 37]. One group has suggested that proficiency in LS generally improves after performing 10–15 cases under supervision, indicating that technical experience can offset anatomic challenges [17]. Thus, it might be reasonable to propose that less experienced laparoscopic surgeons avoid performing their first few LS cases in the setting of massive splenomegaly. Moving forward, it would be beneficial to integrate spleen size and weight into operative planning, particularly when considering adjunctive approaches such as hand-assisted laparoscopy or preoperative CT imaging to assess spleen volume [11]. As surgical expertise and technology continue to evolve, practical guidelines should be updated frequently to incorporate splenic size and weight as selection criteria and reflect modern evidence.

Another key outcome is EBL, which is a critical intraoperative concern during pediatric splenectomy due to its association with transfusion requirements and the potential need for conversion to an open approach. In our study, EBL was similar between the LS and OS groups, aligning with previous pediatric studies [10, 33]. However, when restricting the analysis to more recent studies, LS was associated with lower EBL. This may be explained by the fact that although LS is often considered more technically challenging and carries a higher bleeding risk, especially in cases of splenomegaly or limited working space, advances in surgical technique and perioperative care have likely contributed to less bleeding outcomes [38]. For instance, some studies have noted that modern vessel-sealing technologies and optimized port placement can improve vascular control, especially at the hilar and short gastric vessels, thus reducing bleeding even in difficult cases [18, 26]. Moreover, LS provides enhanced visualization that allows for precise dissection and hemostasis [24, 26, 33]. It is also important to consider that EBL can be reported differently across techniques. In LS, intrasplenic blood evacuated during morcellation may artificially elevate recorded blood loss, whereas in OS, this volume is typically removed within the intact specimen [11]. For this reason, some have argued that transfusion rates may more accurately reflect clinically relevant bleeding than EBL alone [25]. In summary, our findings support similar intraoperative bleeding outcomes with emerging evidence that LS may be associated with reduced blood loss in more contemporary practice, while additional outcomes, such as transfusion rate, may be used in pediatric splenectomy procedures to precisely address bleeding risk.

In evaluating surgical safety, overall complications remain an essential outcome. Our analysis showed similar overall complication rates between the LS and OS groups, consistent with existing pediatric literature [2, 25]. However, the types of complications did show some variation. Although postoperative complications such as ACS, ileus, and fever, have been described, we observed that the majority of complications in the LS group were respiratory in nature [15, 18]. This may be attributed to the pneumoperitoneum, which elevates the intra-abdominal pressure and compresses the bases of both lungs, leading to a basal lung collapse [31]. Given that the incidence of individual complications did not differ in our study, these patterns may reflect variations in perioperative care or institutional protocols rather than the surgical approach itself. Therefore, it will be essential to look after these key areas of complications and to plan accordingly.

## Limitations

Despite the strengths of this meta-analysis, several important limitations need to be acknowledged. Firstly, the studies included in this analysis are all retrospective cohort studies, with no RCTs available for comparison. Furthermore, most studies were conducted in a single-center setting, hence analyzing within multicenter studies could improve the sample size and generalizability of this meta-analysis. Secondly, there was notable heterogeneity across studies, particularly regarding surgical experiences, case volume, and institutional protocols. Many studies lacked detailed reporting on surgical expertise and level of specialized centers, making it difficult to adjust for this important confounder. This variability limits the generalizability of findings and could have overestimated true differences between LS and OS. Thirdly, another limitation is the lack of standardized reporting of detailed baseline data and preoperative characteristics in many of the studies included. Without these critical data points, it is difficult to assess the comparability of the groups at baseline, which could influence postoperative outcomes. Moreover, many studies did not perform subgroup analyses based on patient comorbidities, which could further contribute to variability in outcomes. Fourthly, concomitant cholecystectomy was commonly performed as reported in fifteen studies [4, 7, 8, 14, 17, 26–28, 30–36], while only five studies [8, 25, 28, 30, 33] provided data for splenectomy alone. Consequently, reported operative times may reflect combined procedures. This conflation may bias comparisons between LS and OS, as laparoscopy is often favored for combined procedures due to reduced overall morbidity. Finally, several studies exhibited a high risk of bias, including selective outcome reporting and incomplete data, particularly those utilizing large administrative databases or involving multiple contributors. These limitations could compromise the accuracy and reliability of pooled estimates. Long-term outcomes, including recurrence related to accessory spleens or late complications, were rarely assessed, restricting conclusions about sustained efficacy and safety. Future research, in the form of prospective, multicenter RCTs with standardized reporting and long-term follow-up, should be conducted to further clarify the comparative effectiveness of LS versus OS for pediatric hematologic conditions.

## Conclusion

This meta-analysis demonstrated that LS could be used as an alternative to OS for selected pediatric patients with non-traumatic splenic disease. Despite longstanding concerns over splenomegaly and intraoperative bleeding, our findings affirm that LS offers reduced estimated blood loss and perioperative advantages including shorter hospital stays, earlier resumption of oral intake, and reduced transfusion requirements, with comparable overall complications. Given the evolving role of LS in pediatric surgical practice, future efforts should focus on standardizing the criteria of massive splenomegaly and refining perioperative strategies to optimize patient outcomes.

## Supplementary Information

Below is the link to the electronic supplementary material.

Supplementary file1 (DOCX 963 KB)

## Data Availability

No datasets were generated or analysed during the current study.

## Abbreviations

EBL: Estimated blood loss
HALS: Hand-assisted laparoscopic splenectomy
HS: Hereditary spherocytosis
ITP: Idiopathic thrombocytopenic purpura
LS: Laparoscopic splenectomy
MIS: Minimally invasive surgery
NSQIP-P: National surgical quality improvement program-pediatric
OS: Open splenectomy
SAGES: Society of American Gastrointestinal and Endoscopic Surgeons
SCD: Sickle cell disease

## References

[1] Mack SJ, Pace DJ, Patil S, Cooke-Barber J, Boelig MM, Berman L (2024) Concurrent cholecystectomy does not increase splenectomy morbidity in patients with hemolytic anemia: a pediatric NSQIP analysis. J Pediatr Surg 59(1):117–123. 10.1016/j.jpedsurg.2023.09.010 37833213 10.1016/j.jpedsurg.2023.09.010

[2] Varghese S, Akhtarkhavri A, Jensen J, Patra SR, Algud SM (2024) Comparative analysis of laparoscopic splenectomy versus open splenectomy in pediatric patients: a retrospective study. Int J Med Public Health 14(2):588–592. 10.5530/ijmedph.2024.2.113

[3] Rescorla FJ, Engum SA, West KW, Tres Scherer LR, Rouse TM, Grosfeld JL (2002) Laparoscopic splenectomy has become the gold standard in children. Am Surg 68(3):297–301; discussion 301–302. https://pubmed.ncbi.nlm.nih.gov/11894857/11894857

[4] Minkes RK, Lagzdins M, Langer JC (2000) Laparoscopic versus open splenectomy in children. J Pediatr Surg 35(5):699–701. 10.1053/jpsu.2000.6010 10813328 10.1053/jpsu.2000.6010

[5] Konca Ç, Söker M, Taş MA, Yıldırım R (2015) Hereditary spherocytosis: evaluation of 68 children. Indian J Hematol Blood Transfus 31(1):127–132 25548458 10.1007/s12288-014-0379-z PMC4275513

[6] Vasilescu C, Stanciulea O, Tudor S (2012) Laparoscopic versus robotic subtotal splenectomy in hereditary spherocytosis. Potential advantages and limits of an expensive approach. Surg Endosc 26(10):2802–2809. 10.1007/s00464-012-2249-9 22476842 10.1007/s00464-012-2249-9

[7] Moores DC, McKee MA, Wang H, Fischer JD, Smith JW, Andrews HG (1995) Pediatric laparoscopic splenectomy. J Pediatr Surg 30(8):1201–1205. 10.1016/0022-3468(95)90022-5 7472983 10.1016/0022-3468(95)90022-5

[8] Reddy VS, Phan HH, O’Neill JA et al (2001) Laparoscopic versus open splenectomy in the pediatric population: a contemporary single-center experience. Am Surg 67(9):859–864 11565764

[9] Zhu QL, Wu W (2021) Comparison of clinical efficacy of laparoscopic splenectomy versus open splenectomy for idiopathic thrombocytopenic purpura. Medicine 100(4):e24436. 10.1097/md.0000000000024436 33530246 10.1097/MD.0000000000024436 PMC7850653

[10] Koshenkov VP, Németh ZH, Carter MS (2011) Laparoscopic splenectomy: outcome and efficacy for massive and supramassive spleens. Am J Surg 203(4):517–522. 10.1016/j.amjsurg.2011.05.014 21924403 10.1016/j.amjsurg.2011.05.014

[11] Shin RD, Lis R, Levergood NR, Brooks DC, Shoji BT, Tavakkoli A (2018) Laparoscopic versus open splenectomy for splenomegaly: the verdict is unclear. Surg Endosc 33(4):1298–1303. 10.1007/s00464-018-6394-7 30167946 10.1007/s00464-018-6394-7

[12] Delaitre B, Maignien B (1991) Splenectomy by the laparoscopic approach. Report of a case. Presse Med 20(44):2263 1838167

[13] Tulman S, Holcomb GW, Karamanoukian HL, Reynhout J (1993) Pediatric laparoscopic splenectomy. J Pediatr Surg 28(5):689–692. 10.1016/0022-3468(93)90033-h 8340860 10.1016/0022-3468(93)90033-h

[14] Deng XG, Maharjan A, Tang J et al (2012) A modified laparoscopic splenectomy for massive splenomegaly in children with hematological disorder: a single institute retrospective clinical research. Pediatr Surg Int 28(12):1201–1209. 10.1007/s00383-012-3215-2 23184263 10.1007/s00383-012-3215-2

[15] Feng S, Qiu Y, Li X et al (2015) Laparoscopic versus open splenectomy in children: a systematic review and meta-analysis. Pediatr Surg Int 32(3):253–259. 10.1007/s00383-015-3845-2 26661732 10.1007/s00383-015-3845-2

[16] Altaf AMS, Sawatzky M, Ellsmere J et al (2009) Laparoscopic accessory splenectomy: the value of perioperative localization studies. Surg Endosc 23(12):2675–2679. 10.1007/s00464-008-0258-5 19165541 10.1007/s00464-008-0258-5

[17] Esposito C, Corcione F, Garipoli V, Ascione G (1997) Pediatric laparoscopic splenectomy: are there real advantages in comparison with the traditional open approach? Pediatr Surg Int 12(7):509–510. 10.1007/bf01258713 9238118 10.1007/BF01258713

[18] Winslow ER, Brunt LM (2003) Perioperative outcomes of laparoscopic versus open splenectomy: a meta-analysis with an emphasis on complications. Surgery 134(4):647–653. 10.1016/s0039-6060(03)00312-x 14605626 10.1016/s0039-6060(03)00312-x

[19] Moher D, Liberati A, Tetzlaff J, Altman DG, Prisma Group (2009) Preferred reporting items for systematic reviews and meta-analyses: the PRISMA statement. PLoS Med 6(7):E1000097 19621072 10.1371/journal.pmed.1000097 PMC2707599

[20] Sterne JA et al (2016) ROBINS-I: a tool for assessing risk of bias in non-randomised studies of interventions. BMJ 355:i4919 27733354 10.1136/bmj.i4919 PMC5062054

[21] Higgins JP, Thompson SG, Deeks JJ, Altman DG (2003) Measuring inconsistency in meta-analyses. BMJ 327(7414):557–560 12958120 10.1136/bmj.327.7414.557 PMC192859

[22] Higgins JPT, Cochrane Collaboration (2020) Cochrane handbook for systematic reviews of interventions. In: Higgins JPT, Green S (eds) Cochrane book series, 2nd edn. Hoboken, NJ: Wiley-Blackwell.

[23] Esposito C, Luca UD, Cerulo M et al (2022) Twenty-five-year experience with minimally invasive splenectomy in children: from minilaparotomy to use of sealing devices and indocyanine green fluorescence technology: tips and tricks and technical considerations. J Laparoendosc Adv Surg Tech A 32(9):1010–1015. 10.1089/lap.2022.0038 35796697 10.1089/lap.2022.0038

[24] Hassan ME, Al Ali K (2014) Massive splenomegaly in children: laparoscopic versus open splenectomy. JSLS J Soc Laparoendosc Surg 18(3):e2014.00245. 10.4293/jsls.2014.00245 10.4293/JSLS.2014.00245 PMC4154414 25392624

[25] Utria AF, Goffredo P, Keck K, Shelton JS, Shilyansky J, Hassan I (2019) Laparoscopic splenectomy: has it become the standard surgical approach in pediatric patients? J Surg Res 240:109–114. 10.1016/j.jss.2019.02.045 30925411 10.1016/j.jss.2019.02.045

[26] Zhu J, Ye H, Wang Y (2011) Laparoscopic versus open pediatric splenectomy for massive splenomegaly. Surg Innov 18(4):349–353. 10.1177/1553350611400758 21385756 10.1177/1553350611400758

[27] Alwabari A, Parida L, Al-Salem AH (2009) Laparoscopic splenectomy and/or cholecystectomy for children with sickle cell disease. Pediatr Surg Int 25(5):417–421. 10.1007/s00383-009-2352-8 19370356 10.1007/s00383-009-2352-8

[28] Farah RA, Rogers ZR, Thompson WR, Hicks BA, Guzzetta PC, Buchanan GR (1997) Comparison of laparoscopic and open splenectomy in children with hematologic disorders. J Pediatr 131(1):41–46. 10.1016/s0022-3476(97)70122-7 9255190 10.1016/s0022-3476(97)70122-7

[29] Fachin CG, Amado F, Romaniello G et al (2019) Open versus laparoscopic splenectomies in children: a comparative study performed at a public hospital in Brazil. J Laparoendosc Adv Surg Tech A 29(10):1357–1361. 10.1089/lap.2019.0123 31539304 10.1089/lap.2019.0123

[30] Janu PG, Rogers DA, Lobe TE (1996) A comparison of laparoscopic and traditional open splenectomy in childhood. J Pediatr Surg 31(1):109–114. 10.1016/s0022-3468(96)90330-9 8632260 10.1016/s0022-3468(96)90330-9

[31] Khirallah MG, Eldesouki NI, Hasaballah SZ, Elshanshoury M (2017) Laparoscopic versus open splenectomy in children with benign hematological diseases in children. Ann Pediatr Surg 13(4):194–198. 10.1097/01.xps.0000522255.44536.30

[32] Makansi M, Hutter M, Theilen TM, Fiegel HC, Rolle U, Gfroerer S (2021) Comparison of perioperative outcomes between laparoscopic and open partial splenectomy in children and adolescents. World J Gastrointest Surg 13(9):979–987. 10.4240/wjgs.v13.i9.979 34621474 10.4240/wjgs.v13.i9.979 PMC8462087

[33] Qureshi FG, Ergun O, Sandulache VC, et al (2005) Laparoscopic splenectomy in children. JSLS 9(4):389–392. https://pubmed.ncbi.nlm.nih.gov/16381351/PMC3015648 16381351

[34] Waldhausen JH, Tapper D (1997) Is pediatric laparoscopic splenectomy safe and cost-effective? Arch Surg 132(8):822–822. 10.1001/archsurg.1997.01430320024003 9267264 10.1001/archsurg.1997.01430320024003

[35] Li Y, Wang C, Chen W et al (2023) Selection of surgical modality for massive splenomegaly in children. Surg Endosc 37(12):9070–9079. 10.1007/s00464-023-10462-7 37798532 10.1007/s00464-023-10462-7 PMC10709218

[36] Yoshida K, Yamazaki Y, Mizuno R et al (1995) Laparoscopic splenectomy in children. Surg Endosc 9(12):1279–1282. 10.1007/bf00190159 8629209 10.1007/BF00190159

[37] Al-Mulhim AS (2012) Laparoscopic splenectomy for massive splenomegaly in benign hematological diseases. Surg Endosc 26(11):3186–3189. 10.1007/s00464-012-2314-4 22580880 10.1007/s00464-012-2314-4

[38] Somasundaram S, Massey L, Gooch D, Reed J, Menzies D (2015) Laparoscopic splenectomy is emerging “gold standard” treatment even for massive spleens. Ann R Coll Surg Engl 97(5):345–348. 10.1308/003588414x14055925060479 26264084 10.1308/003588414X14055925060479 PMC5096558

