# Supplementary material for: Laparoscopic versus open splenectomy in non-traumatic pediatric patients: a systematic review and meta-analysis
Source: Pediatr Surg Int. 2025 Oct 17;41(1):317. doi: 10.1007/s00383-025-06208-2 (PMC12534274; doi:10.1007/s00383-025-06208-2)

**Supplemental Item 1: Search Strategy**

**Medline**

Ovid MEDLINE(R) ALL <1946 to January 29, 2025>

1 Minimally Invasive.ti,ab. 100201

2 Minimally Invasive Surgical Procedures/ 31518

3 Laparoscopy/ 109631

4 Laparoscop*.ti,ab. 163226

5 Endoscopy/ 62153

6 Endoscop*.ti,ab. 267752

7 ((endoscop* or Laparoscop*) and splenectomy).ti,ab. 3002

8 Minimally Invasive splenectomy.ti,ab. 18

9 1 or 2 or 3 or 4 or 5 or 6 or 7 or 8 525591

10 open surg*.ti,ab. 33922

11 Conversion to Open Surgery/ 1485

12 open Splenomegaly.ti,ab. 1

13 open splenectomy.ti,ab. 398

14 open partial splenectomy.ti,ab. 14

15 10 or 11 or 12 or 13 or 14 35198

16 splenectomy.ti,ab. 22706

17 Splenectomy/ 22913

18 (splenomegaly or spleen).ti,ab. 164875

19 Splenomegaly/ 11212

20 partial splenectomy.ti,ab. 616

21 16 or 17 or 18 or 19 or 20 192610

22 Treatment Outcome/ 1230626

23 (surgical and (outcome or modality)).ti,ab. 180237

24 (perioperative and (complication or outcome*)).ti,ab. 64782

25 Postoperative Complications/ 419599

26 Postoperative Complications*.ti,ab. 84911

27 Post-operative Complications*.ti,ab. 9509

28 (peri-operative and (complication or outcome*)).ti,ab. 4669

29 Perioperative Care/ or Perioperative Period/ 21024

30 length of stay.ti,ab. 88584

31 "Length of Stay"/ 108795

32 operative time.ti,ab. 40516

33 Operative Time/ 20030

34 surgical wound*.ti,ab. 7610

35 Blood Loss, Surgical/ 21570

36 surgical blood loss.ti,ab. 581

37 22 or 23 or 24 or 25 or 26 or 27 or 28 or 29 or 30 or 31 or 32 or 33 or 34 or 35 or 36 1875112

38 p?ediatric*.ti,ab. 495330

39 Pediatrics/ 59406

40 38 or 39 510885

41 9 and 15 and 21 and 37 and 40 37

**Embase**

Embase <1974 to 2025 January 29>

1 Minimally Invasive.ti,ab. 146772

2 minimally invasive surgery/ 59663

3 laparoscopy/ 101848

4 Laparoscop*.ti,ab. 263256

5 endoscopy/ 142686

6 Endoscop*.ti,ab. 433984

7 ((endoscop* or Laparoscop*) and splenectomy).ti,ab. 4871

8 Minimally Invasive splenectomy.ti,ab. 26

9 1 or 2 or 3 or 4 or 5 or 6 or 7 or 8 840160

10 open surg*.ti,ab. 51357

11 open surgery/ 27395

12 open partial splenectomy.ti,ab. 16

13 open Splenomegaly.ti,ab. 1

14 10 or 11 or 12 or 13 66418

15 splenectomy.ti,ab. 30215

16 partial splenectomy/ or splenectomy/ 42519

17 splenomegaly/ 43496

18 (splenomegaly or spleen).ti,ab. 212780

19 partial Splenectomy.ti,ab. 790

20 15 or 16 or 17 or 18 or 19 263552

21 treatment outcome/ 1016854

22 (surgical and (outcome or modality)).ti,ab. 271095

23 (perioperative and (complication or outcome*)).ti,ab. 98240

24 postoperative complication/ 442343

25 Postoperative Complications*.ti,ab. 118452

26 Post-operative Complications*.ti,ab. 20359

27 (peri-operative and (complication or outcome*)).ti,ab. 11364

28 perioperative complication/ or perioperative care/ or perioperative period/ 76088

29 length of stay.ti,ab. 165252

30 "length of stay"/ 302125

31 operative time.ti,ab. 69254

32 operation duration/ 164162

33 surgical wound*.ti,ab. 9862

34 operative blood loss/ 43359

35 surgical blood loss.ti,ab. 826

36 21 or 22 or 23 or 24 or 25 or 26 or 27 or 28 or 29 or 30 or 31 or 32 or 33 or 34 or 35 2034518

37 9 and 14 and 20 and 36 625

38 open splenectomy.ti,ab. 526

39 14 or 38 66858

40 9 and 20 and 36 and 39 895

41 p?ediatric*.ti,ab. 771641

42 pediatrics/ 96210

43 41 or 42 798527

44 40 and 43 68

**CINAHL**

| # | Query | Last Run Via | Results |
| --- | --- | --- | --- |
| S35 | S8 AND S13 AND S19 AND S34 | Interface - EBSCOhost Research Databases Search Screen - Advanced Search Database - CINAHL | 64 |
| S34 | S20 OR S21 OR S22 OR S23 OR S24 OR S25 OR S26 OR S27 OR S28 OR S29 OR S30 OR S31 OR S32 OR S33 | Interface - EBSCOhost Research Databases Search Screen - Advanced Search Database - CINAHL | 619,559 |
| S33 | TI surgical blood loss OR AB surgical blood loss | Interface - EBSCOhost Research Databases Search Screen - Advanced Search Database - CINAHL | 133 |
| S32 | (MH "Blood Loss, Surgical") | Interface - EBSCOhost Research Databases Search Screen - Advanced Search Database - CINAHL | 6,299 |
| S31 | TI surgical wound* OR AB surgical wound* | Interface - EBSCOhost Research Databases Search Screen - Advanced Search Database - CINAHL | 1,910 |
| S30 | TI operative time OR AB operative time | Interface - EBSCOhost Research Databases Search Screen - Advanced Search Database - CINAHL | 7,799 |
| S29 | (MH "Length of Stay") | Interface - EBSCOhost Research Databases Search Screen - Advanced Search Database - CINAHL | 53,350 |
| S28 | TI length of stay OR AB length of stay | Interface - EBSCOhost Research Databases Search Screen - Advanced Search Database - CINAHL | 34,896 |
| S27 | (MH "Perioperative Care") | Interface - EBSCOhost Research Databases Search Screen - Advanced Search Database - CINAHL | 13,495 |
| S26 | TI peri-operative and (complication* or outcome*) OR AB peri-operative and (complication* or outcome*) | Interface - EBSCOhost Research Databases Search Screen - Advanced Search Database - CINAHL | 1,659 |
| S25 | TI Post-operative Complications* OR AB Post-operative Complications* | Interface - EBSCOhost Research Databases Search Screen - Advanced Search Database - CINAHL | 1,699 |
| S24 | TI Postoperative Complications* OR AB Postoperative Complications* | Interface - EBSCOhost Research Databases Search Screen - Advanced Search Database - CINAHL | 14,318 |
| S23 | (MH "Postoperative Complications") | Interface - EBSCOhost Research Databases Search Screen - Advanced Search Database - CINAHL | 85,838 |
| S22 | TI perioperative and (complication* or outcome*) OR AB perioperative and (complication* or outcome*) | Interface - EBSCOhost Research Databases Search Screen - Advanced Search Database - CINAHL | 21,798 |
| S21 | TI ( surgical and (outcome or modality) ) OR AB ( surgical and (outcome or modality) ) | Interface - EBSCOhost Research Databases Search Screen - Advanced Search Database - CINAHL | 76,363 |
| S20 | (MH "Treatment Outcomes") | Interface - EBSCOhost Research Databases Search Screen - Advanced Search Database - CINAHL | 443,844 |
| S19 | S14 OR S15 OR S16 OR S17 OR S18 | Interface - EBSCOhost Research Databases Search Screen - Advanced Search Database - CINAHL | 11,392 |
| S18 | TI partial splenectomy OR AB partial splenectomy | Interface - EBSCOhost Research Databases Search Screen - Advanced Search Database - CINAHL | 69 |
| S17 | (MH "Splenomegaly") | Interface - EBSCOhost Research Databases Search Screen - Advanced Search Database - CINAHL | 1,064 |
| S16 | TI ( splenomegaly or spleen ) OR AB ( splenomegaly or spleen ) | Interface - EBSCOhost Research Databases Search Screen - Advanced Search Database - CINAHL | 8,841 |
| S15 | (MH "Splenectomy") | Interface - EBSCOhost Research Databases Search Screen - Advanced Search Database - CINAHL | 1,858 |
| S14 | TI splenectomy OR AB splenectomy | Interface - EBSCOhost Research Databases Search Screen - Advanced Search Database - CINAHL | 2,237 |
| S13 | S9 OR S10 OR S11 OR S12 | Interface - EBSCOhost Research Databases Search Screen - Advanced Search Database - CINAHL | 5,530 |
| S12 | TI open partial splenectomy OR AB open partial splenectomy | Interface - EBSCOhost Research Databases Search Screen - Advanced Search Database - CINAHL | 2 |
| S11 | TI open splenectomy OR AB open splenectomy | Interface - EBSCOhost Research Databases Search Screen - Advanced Search Database - CINAHL | 63 |
| S10 | TI open Splenomegaly OR AB open Splenomegaly | Interface - EBSCOhost Research Databases Search Screen - Advanced Search Database - CINAHL | 0 |
| S9 | TI open surg* OR AB open surg* | Interface - EBSCOhost Research Databases Search Screen - Advanced Search Database - CINAHL | 5,473 |
| S8 | S1 OR S2 OR S3 OR S4 OR S5 OR S6 OR S7 | Interface - EBSCOhost Research Databases Search Screen - Advanced Search Database - CINAHL | 112,339 |
| S7 | TI Minimally Invasive splenectomy OR AB Minimally Invasive splenectomy | Interface - EBSCOhost Research Databases Search Screen - Advanced Search Database - CINAHL | 5 |
| S6 | TI ( (endoscop* or Laparoscop*) and splenectomy ) OR AB ( (endoscop* or Laparoscop*) and splenectomy ) | Interface - EBSCOhost Research Databases Search Screen - Advanced Search Database - CINAHL | 493 |
| S5 | TI Endoscop* OR AB Endoscop* | Interface - EBSCOhost Research Databases Search Screen - Advanced Search Database - CINAHL | 54,689 |
| S4 | TI Laparoscop* OR AB Laparoscop* | Interface - EBSCOhost Research Databases Search Screen - Advanced Search Database - CINAHL | 33,567 |
| S3 | (MH "Laparoscopy") OR (MH "Surgery, Laparoscopic") | Interface - EBSCOhost Research Databases Search Screen - Advanced Search Database - CINAHL | 23,809 |
| S2 | (MH "Minimally Invasive Procedures") | Interface - EBSCOhost Research Databases Search Screen - Advanced Search Database - CINAHL | 13,738 |
| S1 | TI Minimally Invasive OR AB Minimally Invasive | Interface - EBSCOhost Research Databases Search Screen - Advanced Search Database - CINAHL | 20,293 |

**Cochrane Library**

#1 (Minimally Invasive):ti,ab,kw 9383

#2 MeSH descriptor: [Minimally Invasive Surgical Procedures] this term only 1467

#3 MeSH descriptor: [Laparoscopy] this term only 7516

#4 (Laparoscop*):ti,ab,kw 29982

#5 MeSH descriptor: [Endoscopy] this term only 2626

#6 (Endoscop*):ti,ab,kw 36914

#7 ((endoscop* or Laparoscop*) and splenectomy):ti,ab,kw 135

#8 (Minimally Invasive splenectomy):ti,ab,kw 21

#9 #1 or #2 or #3 or #4 or #5 or #6 or #7 or #8 71632

#10 (open surg*):ti,ab,kw 32539

#11 MeSH descriptor: [Conversion to Open Surgery] this term only 65

#12 (open Splenomegaly):ti,ab,kw 186

#13 (open splenectomy):ti,ab,kw 175

#14 (open partial splenectomy):ti,ab,kw 12

#15 #10 or #11 or #12 or #13 or #14 32767

#16 MeSH descriptor: [Treatment Outcome] this term only 197835

#17 (surgical and (outcome or modality)):ti,ab,kw 69606

#18 (perioperative outcome*):ti,ab,kw 15997

#19 MeSH descriptor: [Postoperative Complications] this term only 24057

#20 (Postoperative Complications*):ti,ab,kw 61706

#21 (Post-operative Complications*):ti,ab,kw 8655

#22 (peri-operative outcome*):ti,ab,kw 1583

#23 (peri-operative complication*):ti,ab,kw 1161

#24 MeSH descriptor: [Perioperative Care] this term only 1374

#25 MeSH descriptor: [Perioperative Period] this term only 384

#26 (length of stay):ti,ab,kw 37127

#27 MeSH descriptor: [Length of Stay] explode all trees 9749

#28 (operative time):ti,ab,kw 27867

#29 MeSH descriptor: [Operative Time] this term only 2261

#30 (surgical wound*):ti,ab,kw 17636

#31 MeSH descriptor: [Blood Loss, Surgical] this term only 3637

#32 (surgical blood loss):ti,ab,kw 11468

#33 #16 or #17 or #18 or #19 or #20 or #21 or #22 or #23 or #24 or #25 or #26 or #27 or #28 or #29 or #30 or #31 or #32 333899

#34 (splenectomy):ti,ab,kw 832

#35 MeSH descriptor: [Splenectomy] this term only 252

#36 (splenomegaly or spleen):ti,ab,kw 4405

#37 MeSH descriptor: [Splenomegaly] this term only 106

#38 (partial splenectomy):ti,ab,kw 71

#39 #34 or #35 or #36 or #37 or #38 5049

#40 #9 and #15 and #33 and #39 91

**Manually searched and added**

| 1 | Makansi et al Comparison of perioperative outcomes between laparoscopic and open partial splenectomy in children and adolescents (2021) |
| --- | --- |
| 2 | Khirallah et al Laparoscopic versus open splenectomy in children with benign hematological diseases in children. (2017) |
| 3 | Minkes et al Laparoscopic versus open splenectomy in children |
| 4 | Fachin et al. Open Versus Laparoscopic Splenectomies in Children: A Comparative Study Performed at a Public Hospital in Brazil (2019) |

**Supplemental Item 2: Risk of Bias in Non-Randomized Studies of Interventions (ROBINS-I)**


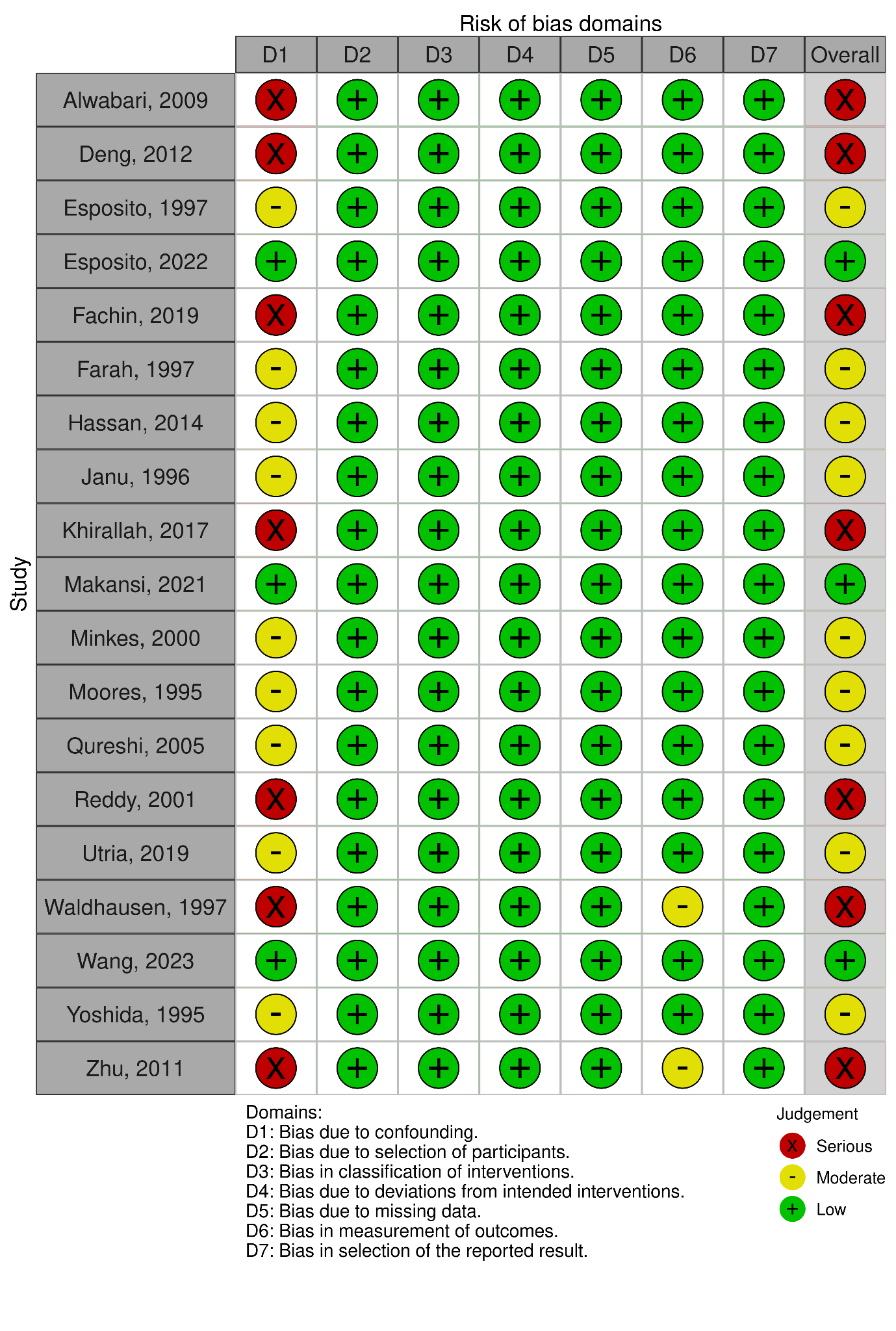


**Supplemental Item 3: PRISMA flowchart of the study selection process**


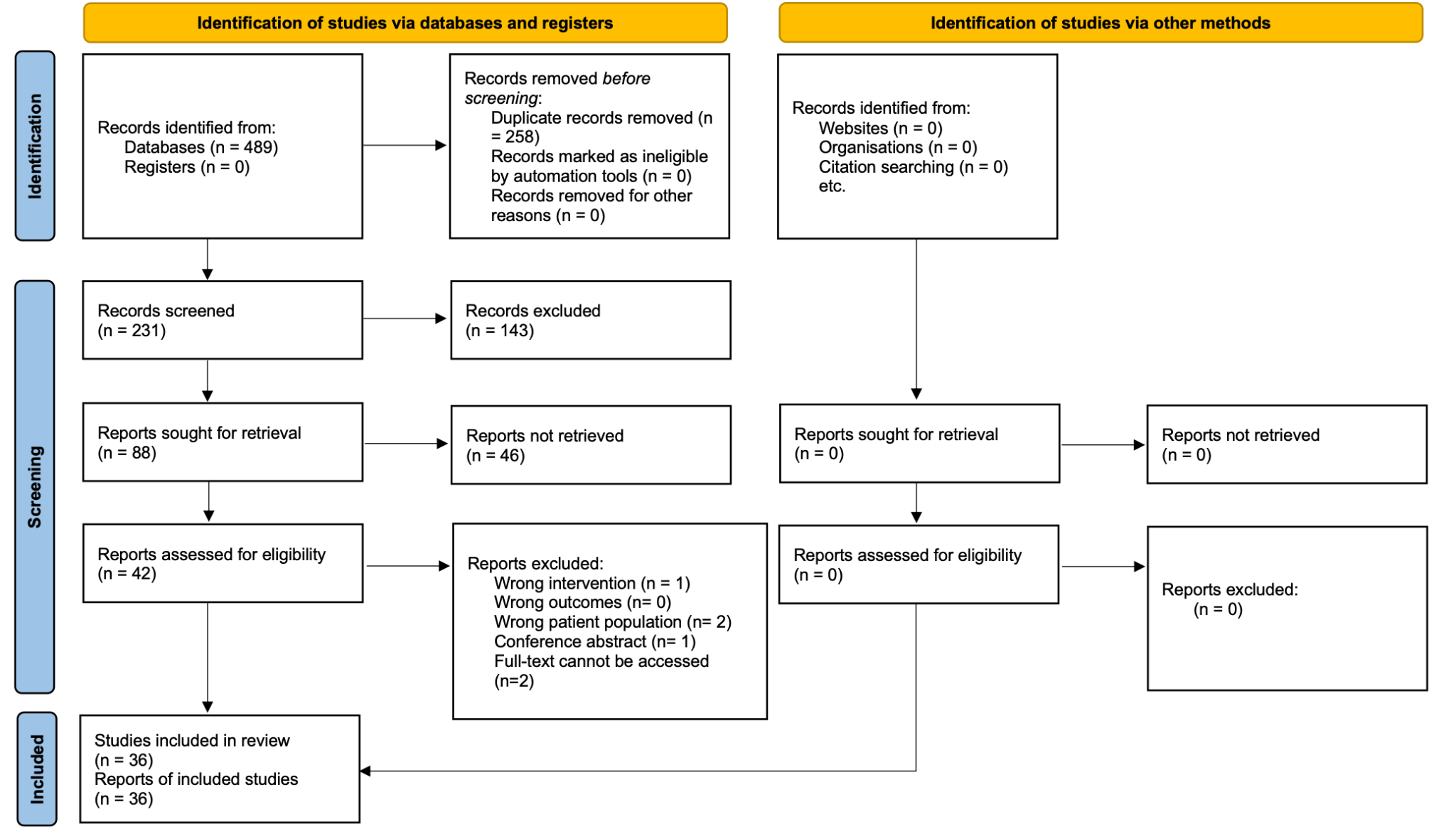

Supplement: Supplementary file 1 — Supplementary file1 (DOCX 963 KB) [file 383_2025_6208_MOESM1_ESM.docx]
